# Retraction: LncRNA-AF113014 promotes the expression of Egr2 by interaction with miR-20a to inhibit proliferation of hepatocellular carcinoma cells

**DOI:** 10.1371/journal.pone.0340233

**Published:** 2026-01-07

**Authors:** The PLOS One Editors

Following the publication of this article [1] concerns were raised with the cell lines and primers reportedly used in this study. Specifically,

- The following four (out of five) cell lines reported in this study have been identified as either contaminated or misclassified:
  - The L02 cell line reported as a human immortalized normal hepatocyte cell line in this study [1] is a contaminated cell line that was previously shown to be a HeLa derivative instead [2].
  - The SMMC7721 cell line reported as a human hepatocellular carcinoma cell line in this study [1] is a contaminated cell line that was previously shown to be a HeLa derivative instead [2,3].
  - The HepG2 cell line reported as a human hepatocellular carcinoma cell line in this study [1] is a misclassified cell line that was previously shown to originate from a hepatoblastoma instead [4].
  - The SK-Hep1 cell line reported as a human hepatocellular carcinoma cell line in this study [1] is a misclassified cell line that was previously shown to originate from an endothelial origin instead [5,6].
- The snRNA primers for U6 (forward and reverse) reported in Table 1 of this article [1] do not appear to target U6.

Corresponding author, HT, stated that the research group was not aware of the cell line concerns previously and that STR profiling was not conducted on the cell lines used in this study. Furthermore, they stated that the research group did not design or validate the primers used in this study and instead used primer sequences previously reported in published articles. The individual-level data underlying Fig 7A were provided for editorial review, but the individual-level data underlying the remaining graphs presented in this article are no longer available.

In light of the above concerns which call into question the reliability of the published results, the *PLOS One* Editors retract this article.

HT did not agree with the retraction and stands by the article’s findings. TZ, DW, JC, YT, XC, HP, LZ, and AH either did not respond directly or could not be reached.

In addition to the above concerns, the *PLOS One* Editors are also aware of similarities between results presented in Fig 2C of this article [1] and results presented in Fig 5B of [7]. PLOS notes that [7] was published at a later date by researchers who do not appear to share affiliations with the authors of [1].
